# Infection of groundnut ringspot virus in *Plumeria pudica* characterized by irregular virus distribution and intermittent expression of symptoms

**DOI:** 10.3389/fpls.2023.1202139

**Published:** 2023-07-26

**Authors:** Gabriel Madoglio Favara, Felipe Franco de Oliveira, Camila Geovana Ferro, Heron Delgado Kraide, Eike Yudi Nishimura Carmo, Vinicius Henrique Bello, Marcos Roberto Ribeiro-Junior, Renate Krause-Sakate, Elliot Watanabe Kitajima, Jorge Alberto Marques Rezende

**Affiliations:** ^1^ Laboratory of Plant Virology, Department of Plant Pathology and Nematology, Escola Superior de Agricultura Luiz de Queiroz, University of São Paulo, Piracicaba, Brazil; ^2^ Laboratory of Plant Virology and Virus-Vector-Host Interactions, Department of Plant Protection, Faculdade de Ciências Agronômicas, São Paulo State University, Botucatu, Brazil

**Keywords:** *Orthotospovirus*, GRSV, virus movement, virus resistance, bridal bouquet

## Abstract

*Plumeria pudica*, known as bridal bouquet, exhibiting characteristic symptoms of orthotospovirus infection were found in different localities in Brazil. Symptoms were restricted to leaves of the middle and lower thirds of a few branches of each plant. Electron microscopy, molecular analyses, and complete genome sequencing identified the orthotospovirus as groundnut ringspot virus (GRSV),member of the species *Orthotospovirus arachianuli*. The virus was poorly transmitted mechanically to *P. pudica*. Reverse transcription polymerase chain reaction (RT-PCR) and reverse transcription quantitative polymerase chain reaction (RT-qPCR) analyses performed using total RNA extracted from leaf blades, primary veins, petioles, and regions of petiole insertion on branches indicated the presence of GRSV, predominantly in the symptomatic leaf blades. Symptomatic branches propagate vegetatively, often resulting in plants expressing GRSV symptoms. In contrast, vegetative propagation of the asymptomatic branches of infected plants predominantly generates plants without GRSV symptoms. The resistance of *P. pudica* plants to GRSV infection, restricted systemic viral movement, and expression of symptoms in infected plants suggest that this orthotospovirus does not threaten this ornamental plant.

## 1 Introduction

Groundnut ringspot virus (GRSV) belongs to the species *Orthotospovirus arachianuli*, genus *Orthotospovirus* and family *Tospoviridae* (Kormelink et al., 2021). The genome of orthotospoviruses comprises three segments of single-stranded RNA. The L (large) segment is negative sense and encodes an RNA-dependent RNA polymerase. The M segment (medium) is ambisense and encodes a nonstructural movement protein and glycoprotein precursors (Gn and Gc). The S (small) segment is ambisense and encodes the nucleocapsid protein (N) and a non-structural silencing suppressor protein (NSs). The average sizes the RNAs are 8.8 kb for L, 4.8 kb for M, and 2.9 kb for S. Orthotospovirus particles are spherical (80–110 nm) and have a lipid envelope where Gn and Gc glycoproteins are inserted; grouping all three RNA molecules (Oliver and Whitfield, 2016; Kormelink et al., 2021). Thrips persistently and propagatively transmit these viruses (Gilbertson et al., 2015).

*Plumeria pudica* Jacq., known as bridal bouquet, is a flowering ornamental plant of the Apocynaceae family native to Colombia, Venezuela, and Panama. It is a shrub with one or two thin trunks that branch close to the ground and form a dense crown. The plants can reach 3–4 m in height and have bright green leaves and white flowers with a yellow center, which have no fragrance. They are widely used as ornamental plants, in medicines, and cosmetics (Choudhary et al., 2014; Chamakuri et al., 2020). In Brazil, *P. pudica* is commonly found in the northeastern region (Fernandes et al., 2015).

To the best of our knowledge, only two tobamoviruses have been reported to infect plants belonging to the genus *Plumeria*. *Plumeria* sp. exhibiting chlorotic rings, mosaics, and leaf deformation were identified as infected with frangipani mosaic virus (FrMV) in Australia in 1971 and India in 1978 (Francki et al., 1971; Varma and Gibbs, 1978). In 2010, *P. rubra* f. *acutifolia* plants coinfected with the tobamovirus FrMV and plumeria mosaic virus (PluMV) were identified in India. The infected plants exhibited mosaic, vein banding, tanning, and necrotic rings (Kumar et al., 2017). In 2017, FrMV was detected in plants of the *Plumeria* sp. in Taiwan (Choliq et al., 2017), and a few years later in *P. obtusa*, *P. rubra*, and *P. stenopetala* plants in the United States (Dey et al., 2020). *P. pudica* plants showing characteristic symptoms of viral infection, such as vein clearing, chlorotic lesions, and concentric rings, were found in the municipality of Piracicaba, state of São Paulo, Brazil. All plants showed the same pattern of symptoms, with a few symptomatic leaves present on only one or two branches of each plant. Here, we present the identification and biological characterization of the Brazilian GRSV isolate infecting *P. pudica*. Experiments were also conducted to better understand the systemic invasion of the virus and expression of symptoms in plants of this species.

## 2 Materials and methods

### 2.1 Plant material

**Figure 1:**
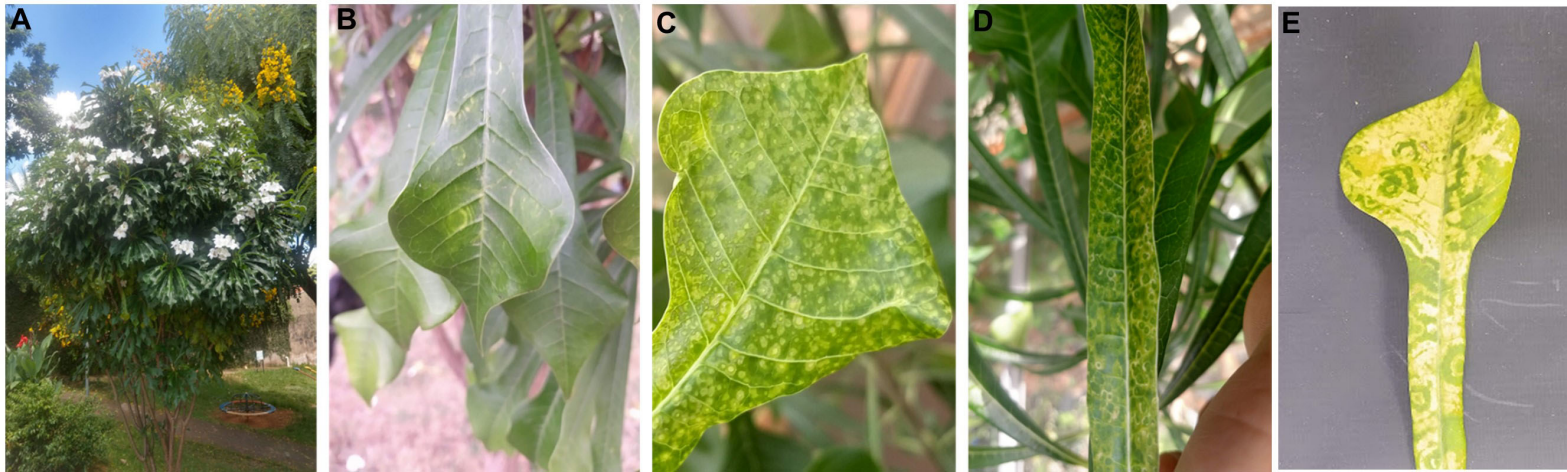
**(A)** *Plumeria pudica* infected with the groundnut ringspot virus (GRSV). **(B–E)** Symptoms of GRSV in some leaves of the plant.

In 2020 and 2021, *P. pudica* plants (**Figure 1A**) located in the municipalities of Piracicaba, São Manuel and Barra Bonita, in the state of São Paulo, Brazil, were inspected in search of symptoms caused by viruses. Six plants exhibited virus-like symptoms on a few leaves (**Figures 1B–E**). Symptomatic and asymptomatic leaves of *P. pudica* were collected and brought to the Laboratory of Plant Virology, Department of Plant Pathology and Nematology, Escola Superior de Agricultura Luiz de Queiroz, University of São Paulo (Esalq/USP), to identify the causal agent.

### 2.2 Transmission electronic microscopy

Fragments of symptomatic leaf lesions were fixed in a modified Karnovsky solution (2.5% glutaraldehyde, 2% formaldehyde in 0.05 M cacodylate buffer, pH 7.2) for 2 h. Subsequently, leaf samples were post-fixed in 2% osmium tetroxide solution, dehydrated in acetone, and embedded in Spurr low-viscosity resin. The blocks were sectioned on a Leica UC6 ultramicrotome, and the obtained ultrathin sections were counterstained with 3% uranyl acetate and 0.5% lead citrate, as previously described by Kitajima and Nome (1999). The presence of viral particles or cytopathological effects in the ultrathin sections of symptomatic leaf tissues was examined under a transmission electron microscope (JEOL JEM 1011).

### 2.3 GRSV detection by reverse transcription polymerase chain reaction and nucleotide sequencing

Total RNA from 21 P*. pudica* plants was separately extracted from the leaf tissues using the Purelink Viral DNA/RNA kit (Thermo Fisher Scientific, Waltham, United States) according to the manufacturer’s recommendations. The extracted RNA was used to detect the virus using universal orthotospovirus BR60/BR65 primers (Eiras et al., 2001), which amplify a 453 bp fragment corresponding to the 3’ untranslated region and a part of the N gene. The amplicons obtained were stained with SYBR Safe DNA Gel Stain (Invitrogen, Eugene, United States), subjected to 1% agarose gel electrophoresis and visualized using a UV light transilluminator. The amplicons were purified using a Wizard SV Gel and PCR Clean-Up System kit (Promega, Madison, United States) and sent for nucleotide sequencing to Macrogen Inc. (South Korea). Nucleotide sequences were compared with corresponding sequences deposited in GenBank using the BLASTn algorithm, available at https://www.ncbi.nlm.nih.gov/blast, to identify the orthotospovirus.

### 2.4 High throughput sequencing

To obtain the complete genome sequence of the GRSV isolate from *P. pudica*, total RNA from a symptomatic plant was extracted using the RNeasy Plant Mini Kit (Qiagen, Hilden, Germany), following the manufacturer’s recommendations. The extracted RNA was sent to Macrogen Inc. (South Korea) for HTS. cDNA libraries were prepared with TruSeq Stranded Total RNA and RiboZero Plant Kit (Illumina, San Diego, United States). Sequencing was performed on an Illumina NovaSeq 6000 platform in the 101-base paired-end reads mode. The raw reads were trimmed with the quality scores limit set to 0.05, ambiguous limit to 2, and minimum number of nucleotides (nt) in reads to 30, using the CLC Genomics Workbench v. 9.0.3 (CLCGW) software. In CLCGW, the trimmed reads were *de novo* assembled to form preliminary contigs using the following parameters: automatic word size, automatic bubble size, minimum contig length 500, map reads back to contigs, match score = 1, mismatch cost = 2, insertion cost = 3, deletion cost = 3, length fraction = 0.5, and similarity fraction = 0.9. The trimmed reads and contigs were imported to Geneious software v.11.1.6. Taxonomic assignment of the contigs was done through BLASTn searches against the virus RefSeq database from NCBI using the Geneious software v.11.1.6. To obtain the complete nucleotide sequences of the L, M, and S segments of the GRSV isolate infecting *P. pudica*, the nucleotide sequences corresponding to L RNA (MH742956), M RNA (MH742957), and S RNA (MH742958) of a GRSV isolate were used as references for mapping HTS data using the Geneious software v.11.1.6. The identities of consensus nucleotide sequences were determined using BLASTn. Phylogenetic relationships were inferred using the Maximum Likelihood method and Tamura-Nei model (Tamura and Nei, 1993), implemented in MEGA 11 (Tamura et al., 2021), with 1000 bootstraps.

### 2.5 GRSV detection by RT-qPCR

A primer pair was designed using the GRSV nucleotide sequences previously obtained in this study for virus detection by RT-qPCR. The GRSV-F (5′-GGTCTACAGTGTTGCACTTTCT-3′) and GRSV-R (5′-CCTTAGACATGGTGGCAGTTT-3′) primers amplify an 80 bp fragment in the gene encoding the nucleocapsid protein. Reactions were performed on the ViiA 7 Real-Time PCR System (Applied Biosystems) using 6.25 µL GoTaq qPCR Master Mix (Promega, Madison, United States), 0.62 µL of GRSV-F and GRSV-R primers (10 µM), 0.19 µL CXR reference dye, 0.25 µL GoScript RT mix for one-step RT-qPCR, 3.32 µL of water, and 1.25 µL of extracted total RNA. Water aliquots were used as non-template controls. Each sample was analyzed in duplicate. The absence of nonspecific amplification was confirmed by melting curve analysis. Samples with a CT score above > 30 were considered negative.

### 2.6 Transmission of GRSV to *P. pudica* plants and partial host range

Symptomatic leaves of *P. pudica* were grounded in 0.02 M phosphate buffer (pH 7), and the extract was mechanically inoculated into leaves of 20 healthy (asymptomatic) *P. pudica* plants and plants of the following species: *Capsicum annuum* (cvs. Amarelo SF 34, Dahra R, Magali, and Quadrado Vermelho), *Chenopodium quinoa*, *Datura stramonium*, *Helianthus annuus* cv. Amarelo alto, *Nicandra physalodes*, *Nicotiana tabacum* cv. Turkish, *Physalis peruviana*, and *Solanum lycopersicum* cv. Santa Clara. Plants of the respective species were inoculated with an isolate of GRSV from tomatoes (control) (**Table 1**). The leaves of the plants were previously sprinkled with carborundum, and the inoculum was applied with the indicator. After two months, 20 P*. pudica* plants were re-inoculated. The inoculum was applied to plants using a toothbrush to generate greater friction on the leaves.

**Table 1:** Reaction of plants inoculated with GRSV isolates from *P. pudica*, tomato, and sweet pepper.

Species/cultivar	No. of positive plants/No of inoculated plants				
	Inoculated with finger and toothbrush		Inoculated with spray gun		
	GRSV isolate		GRSV isolate		
	*P. pudica*	Tomato	*P. pudica*	Tomato	Sweet pepper
*Capsicum annuum* cv. Amarelo SF 134	0/2	1/2	–	–	–
*C. annuum* cv. Dahra R	0/4	4/4	–	–	–
*C. annuum* cv. Magali	0/2	2/2	–	–	–
*C. annuum* cv. Quadrado Vermelho	0/2	1/2	–	–	–
*Chenopodium quinoa*	0/3	2/3	–	–	–
*Datura stramonium*	1/8	2/2	–	–	–
*Helianthus annuus*	0/3	0/2	–	–	–
*Nicandra physalodes*	0/4	0/4	–	–	–
*Nicotiana tabacum* cv. Turkish	1/8	–	–	–	–
*Physalis peruviana*	0/8	4/4	–	–	–
*Plumeria pudica*	0/20	–	1/3	0/6	0/6
*Solanum lycopersicum* cv. Santa Clara	0/5	4/4	–	–	–

-: not inoculated. Detection of GRSV in the inoculated plants was performed by RT-PCR.

Subsequently, 15 P*. pudica* plants were separately inoculated with GRSV isolates from *P. pudica*, tomato, and sweet pepper. The leaf extracts and carborundum were placed inside a container connected to a paint gun and an air compressor (Jet Master II, Schulz) at a constant pressure of 2.8 kg cm^-1^. Inoculation was performed by directing an air jet onto the leaf surfaces of *P. pudica* plants. Three plants were inoculated with the viral isolate from *P. pudica*, and six plants were inoculated with the isolates from tomato and sweet pepper. The presence of GRSV in the inoculated plants was evaluated by RT-PCR.

### 2.7 Systemic distribution of GRSV in *P. pudica* plants

#### 2.7.1 Detection of GRSV in different parts of plants

Symptomatic and asymptomatic leaf samples from the upper, middle, and lower thirds of the branches of one of the *P. pudica* infected plants, named P1 (**Table 2**), were collected for virus detection using RT-PCR and RT-qPCR as described in 2.3 and 2.5, respectively. Subsequently, the presence of the virus was also evaluated in the primary veins, petioles, and region of petiole insertion on the main branch of symptomatic and asymptomatic leaves of P1, P2, P3, and P4 *P. pudica* plants (**Table 3**).

**Table 2:** Detection of groundnut ringspot virus (GRSV) in *Plumeria pudica* plants located in the municipalities of Piracicaba, São Manuel and Barra Bonita, state of São Paulo, Brazil.

Identification	Location	Coordinates	Symptoms*	Virus detected	Genbank acession numbers
P1	Piracicaba	22°43’16.4”S 47°37’22.1”W	VC; LC; R	GRSV	OK539546
P2	Piracicaba	22°43’20”S 47°40’48”W	VC; LC; R	GRSV	OK539547
P3	Piracicaba	22°43’20”S 47°40’48”W	VC; LC; R	GRSV	OM972900
P4	Piracicaba	22°42’43.6”S 47°38’33.0”W	VC; LC; R	GRSV	OK539548
P5	Piracicaba	22°43’16.4”S 47°37’22.1”W	VC; LC; R	GRSV	OM972901
P6	Piracicaba	22°40’25.0”S 47°37’44.2”W	VC; LC; R	GRSV	OM972902
P7	Piracicaba	22°43’21.4”S 47°37’03.0”W	No	–	
P8	Piracicaba	22°43’28.9”S 47°37’19.7”W	No	–	
P9	Piracicaba	22°43’28.9”S 47°37’19.7”W	No	–	
P10	Piracicaba	22°43’28.9”S 47°37’19.7”W	No	–	
P11	Piracicaba	22°43’28.7”S 47°37’12.2”W	No	–	
P12	Piracicaba	22°42’32.0”S 47°38’00.3”W	No	–	
P13	Piracicaba	22°42’32.0”S 47°38’00.3”W	No	–	
P14	Piracicaba	22°43’13.1”S 47°37’24.5”W	No	–	
P15	Piracicaba	22°43’20”S 47°40’48”W	No	–	
P16	Piracicaba	22°43’20”S 47°40’48”W	No	–	
P17	Piracicaba	22°43’20”S 47°40’48”W	No	–	
P18	Piracicaba	22°43’20”S 47°40’48”W	No	–	
P19	Piracicaba	22°43’21.4”S 47°37’03.0”W	No		
P20	São Manuel	22°43’49.6”S 48°36’48.1”W	No	–	
P21	Barra Bonita	22°29’40.3”S 48°32’56.1”W	No	–	

*VC, vein clearing; CL, chlorotic lesions; R, ring spots; -, not detected.

**Table 3:** Detection of GRSV by RT-PCR and RT-qPCR in total RNA extracted from the stem, petiole, primary vein, and leaf blade of symptomatic and asymptomatic leaves of *P. pudica*.

Plant/Branch	Portion analyzed	Leaf symptom	RT-PCR	RT-qPCR (CT*)
P1/B2	Branch 1	Yes	–	- (34)
	Petiole1	Yes	+	+ (20)
	Primary Vein 1	Yes	+	+ (20)
	Leaf blade 1	Yes	+	+ (20)
	Branch 2	No	–	- (33)
	Petiole 2	No	–	- (35)
	Primary Vein 2	No	–	- (31)
	Leaf blade 2	No	–	- (35)
P2/B1	Petiole 1	Yes	–	+ (26)
	Primary Vein 1	Yes	–	+ (25)
	Leaf blade 1	Yes	+	+ (20)
	Petiole 2	No	–	- (35)
	Primary Vein 2	No	–	- (34)
	Leaf blade 2	No	–	- (35)
P3/B1	Petiole 1	Yes	–	+ (28)
	Primary Vein 1	Yes	–	+ (30)
	Leaf blade 1	Yes	+	+ (22)
	Petiole 2	No	–	- (36)
	Primary Vein 2	No	–	- (34)
	Leaf blade 2	No	–	- (33)
P4/B1	Leaf blade	Yes	+	+ (19)

*CT, cycle threshold.The values in bold represent positive samples.

#### 2.7.2 Detection of GRSV in vegetatively propagated branches

Molecular analysis of different tissues of vegetatively propagated branches were conducted to better understand the systemic distribution of GRSV in *P. pudica* plants.

The four branches of the P1 plant infected with GRSV were named B1, B2, B3, and B4 (**Table 4**). When the branches were collected, the number of symptomatic and asymptomatic leaves present on each branch was counted. Molecular analyses were performed to confirm the presence or absence of GRSV in the leaves of the branches selected for vegetative propagation. The branches were rooted in 10 L pots containing a mixture of soil, sand, and manure (1:1:1), autoclaved, and kept for development inside a greenhouse. Symptom expression was evaluated after rooting and vegetative development. Virus distribution in different parts of the plant was determined using RT-PCR and RT-qPCR. Analyses for virus detection were performed on leaves collected from the upper, middle, and lower thirds, and from the roots of each plant. The expression of symptoms in the matrix plant P1 after removing branches with symptomatic leaves was also monitored.

**Table 4:** Detection of GRSV, by RT-PCR and RT-qPCR, in total RNA extracted from symptomatic and asymptomatic leaves and roots of *P. pudica* plants after the second cycle of vegetative propagation.

Plant	Portion analyzed	Leaf symptoms	RT-PCR	RT-qPCR (CT*)
P1/B1 (new sprouting)	Leaves of the lower third of the branch	No	–	nt**
	Leaves of the middle third of the branch	Yes	+	**+ (21)**
	Leaves of the upper third of the branch	No	–	- (35)
	Root		–	nt
P1/B1.1	Leaves of the lower third of the branch	No	–	nt
	Leaves of the middle third of the branch	No	–	- (34)
	Leaves of the upper third of the branch	No	–	- (35)
P1/B1.3	Leaves of the lower third of the branch	No	–	**+ (26)**
	Leaves of the middle third of the branch	Yes	+	**+ (23)**
	Leaves of the upper third of the branch	No	–	- (34)
	Root		–	- (35)
P1/B1.4	Leaves of the lower third of the branch	No	–	- (33)
	Leaves of the middle third of the branch	No	–	- (33)
	Leaves of the upper third of the branch	No	–	- (33)
	Root		–	nt
P1/B1.5	Leaves of the lower third of the branch	No	–	- (38)
	Leaves of the middle third of the branch	No	–	- (38)
	Leaves of the upper third of the branch	No	–	- (38)
	Root		–	nt

*CT, cycle threshold; ** nt, not tested.

One of the *P. pudica* plants obtained from the vegetative propagation of branches of the P1 plant was selected for a new cycle of vegetative propagation and was named P1/B1. All branches of this plant were cut and placed in pots for rooting, as described above. The parent plant (P1) was pruned at its base and maintained in the greenhouse for regrowth. After development, symptom expression was evaluated, and the distribution of the virus was determined as previously described.

## 3 Results

### 3.1 Transmission electronic microscopy

**Figure 2:**
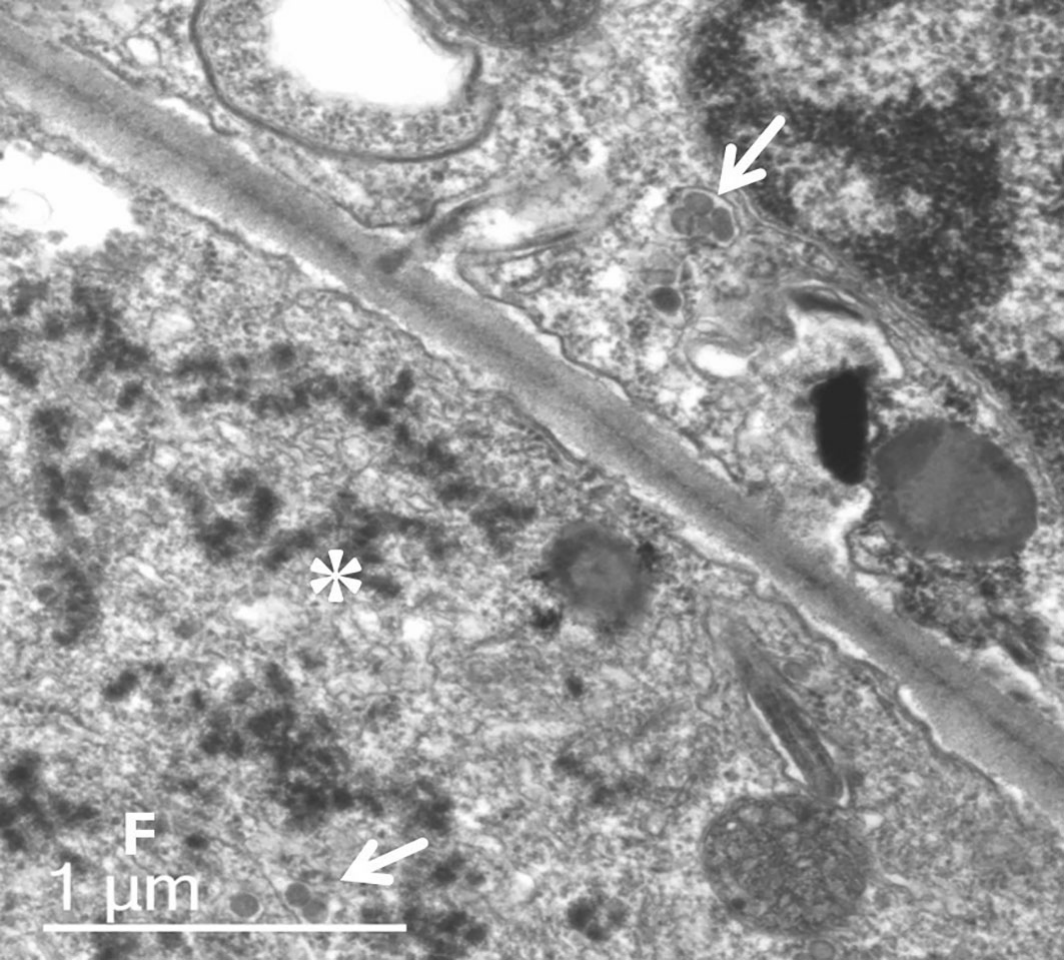
Ultrathin section transmission electron micrograph of symptomatic leaf tissue of *P. pudica*. Typical orthotospovirus particles (arrows) occur at low frequency in the lumen of the endoplasmic reticulum. In most cells, it is possible to observe aggregates of dense masses (*), possibly viral nucleocapsids, which have not completed morphogenesis (membrane involvement). Fibrillar material (F) by the accumulation of non-structural silencing suppressor protein.

Analysis of ultrathin sections of symptomatic leaf tissues revealed the presence of typical orthotospovirus particles at a low frequency in the lumen of the endoplasmic reticulum (**Figure 2**). In the cytoplasm of most cells, aggregates of dense masses were observed, possibly viral nucleocapsids that did not complete morphogenesis; that is, the involvement of the membrane via the Golgi complex. The presence of fibrillar material in the cytoplasm due to the accumulation of NSs was also verified (**Figure 2**).

### 3.2 Detection and identification of GRSV infecting *P. pudica* plants

RT-PCR analyses confirmed the presence of orthotospovirus in six of the 21 P*. pudica* plants evaluated (**Table 2**). The partial nucleotide sequences obtained for the N gene showed 98.42% to 100% identity with the corresponding nucleotide sequence of a GRSV isolate (GenBank accession no. KY400110) identified in infected peanut plants in Brazil. The nucleotide sequences of the identified orthotospoviruses infecting *P. pudica* plants were deposited in GenBank and the accession numbers are listed in **Table 2**. Another ten *P. pudica* plants were inspected in Piracicaba, 45 in Barretos, São Paulo, seven in Londrina, Paraná, and 13 in Lucas do Rio Verde, Mato Grosso. None of the inspected plants exhibited leaves with characteristic symptoms of viral infection and tested negative by RT-PCR.

### 3.3 Complete genome sequence of GRSV isolate from *P. pudica*

A total of 47,862,488 reads were generated for the total RNA extracted from symptomatic leaves of *P. pudica* plant. After trimming and adapter sequence removal, 45,618,008 (average length: 145.9) paired reads were retained. After *de novo* assembles of the trimmed reads 3852 contigs were generated and two contigs with 8876 nt and 3068 nt shared, respectively, 97.6% and 98.3% identity with a GRSV isolate. The complete nucleotide sequences of segments L, M, and S, with mean coverage depths of 9507.9, 8479.5, and 8688.5×, respectively, were determined after mapping the trimmed reads to the GRSV reference sequences (**Supplementary Figure 1**). The L (GenBank OQ656765), M (GenBank OQ656766), and S (GenBank OQ656767) segments were, 8876, 4944 and 3035 nucleotides, respectively, and shared 98.54%, 97.67%, and 97.49% identity with the corresponding nucleotide sequences of a GRSV isolate (GenBank KY350136.1, KY350137.1, and KY400110, respectively) identified in infected peanut plants in Brazil.

The L RNA of GRSV from *P. pudica* has a single open reading frame (ORF) of 8625 nt in length, which encodes a putative 2874 amino acid (aa) RdRp protein. The M RNA contains two ORFs, one with 912 nt corresponding to the NSm gene and another with 3405 nt corresponding to the GnGc gene, resulting in putative encoded proteins of 303 aa and 1134 aa, respectively. The S RNA has two ORFs, one with 1404 nt for the NSs gene and another with 777 nt for the N gene, resulting in encoded putative proteins of 467 aa and 258 aa, respectively.

**Figure 3:**
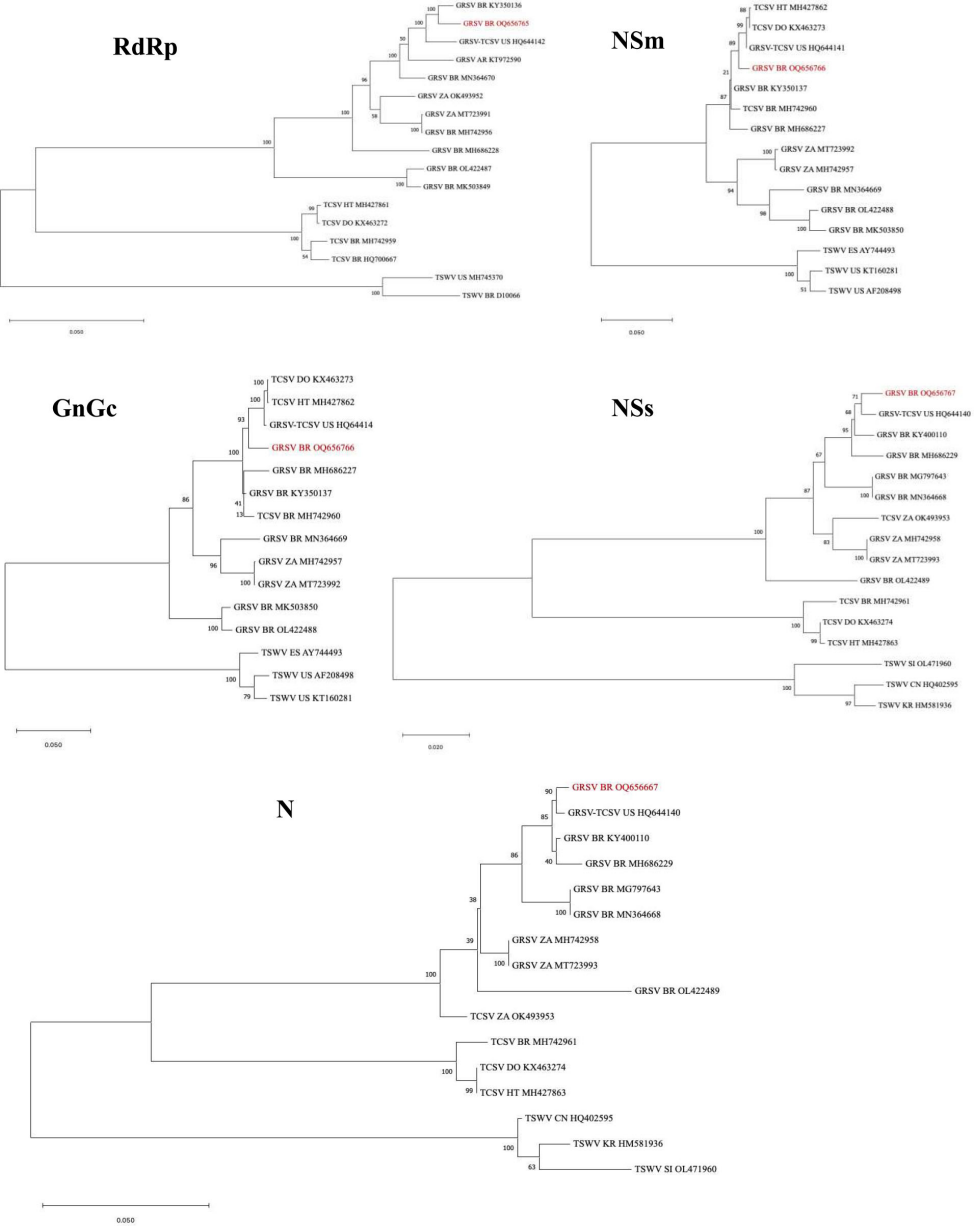
Phylogenetic trees constructed with the complete sequences of the RdRp, NSm, GnGc, NSs, and N genes of the orthotospoviruses groundnut ringspot virus (GRSV), tomato chlorotic virus (TCSV), and tomato spotted wilt virus (TSWV). The isolate of GRSV from *Plumeria pudica* is highlighted in each tree in red. GRSV-TCSV corresponds to the reassortant L_G_M_T_S_G._ Country abbreviations: Brazil (BR); United States (US); Argentina (AR); South Africa (ZA); China (CN); Haiti (HT); Dominican Republic (DO), Slovenia (SI); South Korea (KR); Spain (ES). The corresponding GenBank accession number of each virus sequence is given in the figure. Bar = number of substitutions per site.

In the phylogenetic analysis conducted using the complete nucleotide sequence of the RdRp gene, the GRSV isolate from *P. pudica* was found to be closely related to the GRSV isolate (GenBank KY350136) identified in peanut plants in Brazil (**Figure 3**). In the analyses performed with the complete sequences of the NSm, GnGc, NSs, and N genes, the GRSV isolate from *P. pudica* exhibited a close relationship with the isolate (GenBank HQ644142) identified in infected tomato plants (**Figure 3**). This isolate was the result of a reassortment event between GRSV and TCSV that has the L and S segments coming from GRSV and the M segment from TCSV (Webster et al., 2011).

### 3.4 Transmission of GRSV to *P. pudica* plants and partial host range

The GRSV isolate from *P. pudica* infected one plant of *D. stramonium* and another of *N. tabacum* cv. Turkish (**Table 1**). Both showed local chlorotic lesions on the inoculated leaves. Leaves of *N. tabacum* cv. Turkish also developed concentric rings. None of the plants of the other species were infected with the GRSV isolate of *P. pudica*, including the 20 *P. pudica* plants inoculated mechanically using an indicator or toothbrush. Tomato, sweet pepper, and *P. peruviana* plants inoculated with the GRSV isolate from tomato were infected and showed systemic symptoms of mosaic, and concentric rings. *D. stramonium* and *C. quinoa* exhibited local chlorotic lesions on leaves inoculated with the tomato GRSV isolate. The infection of these plants was confirmed by the observation of symptoms and RT-PCR (**Table 1**).

One of the three *P. pudica* plants inoculated with the GRSV isolate from *P. pudica* (P1) was infected with the inoculum using a spray gun. This plant had only one branch and exhibited symptoms on two leaves in the upper third of the plant. RT-PCR analysis of RNA extracted from the leaves located in the upper, middle, and lower thirds and roots of this plant confirmed the presence of GRSV only in the symptomatic leaves (data not shown). None of the six *P. pudica* plants inoculated with GRSV isolates from tomato or sweet pepper were infected (**Table 1**).

### 3.5 Systemic distribution of GRSV in *P. pudica* plants

#### 3.5.1 Detection of GRSV in different parts of plants

RT-PCR and RT-qPCR detected GRSV in the three symptomatic leaves of P1, regardless of the leaf position on the branch. The samples collected from nine asymptomatic leaves tested negative for the virus. Positive samples had CT ranging from 18 to 20, while negative samples from 34 to 35 (data not shown).

RT-PCR analysis performed with RNA extracted from leaf blades, primary veins, petioles, and regions of petiole insertion on branches indicated the presence of GRSV only in symptomatic leaf blades (**Table 3**). RT-PCR detected the virus in only one of the petiole and primary vein samples tested (**Table 3**). However, RT-qPCR analysis confirmed the presence of the virus in the petiole and primary vein of all symptomatic leaves evaluated (**Table 3**). GRSV was not detected at the petiole insertion sites in the branches of *P. pudica* (**Table 3**). The CT values ​​obtained for the positive samples ranged from 19 to 26, and those for the negative samples from 31 to 36 (**Table 3**).

#### 3.5.2 Detection of GRSV in vegetatively propagated branches

**Figure 4:**
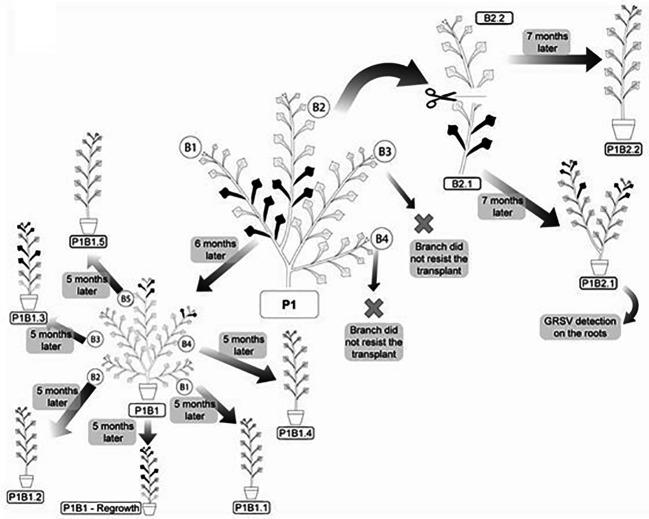
Systemic distribution and symptoms of GRSV in vegetatively propagated branches of *P. pudica*. Symptomatic and asymptomatic leaves are represented by the black and gray colors, respectively.

Four branches (two symptomatic and two asymptomatic) of the P1 plant of *P. pudica* were selected to detect GRSV after vegetative propagation (**Figure 4**). On branch 1 (P1/B1), there were 60 leaves without symptoms and 9 leaves with symptoms. Branch 2 (P1/B2) had 48 leaves without symptoms, and only four had symptoms. This branch was later cut in half to separately verify vegetative propagation of the lower part of the branch (P1/B2.1), which contained symptomatic and asymptomatic leaves, and the upper part of the branch (P1/B2.2), which contained only asymptomatic leaves (**Figure 4**). Branch 3 (P1/B3) had 20 asymptomatic leaves, and branch 4 (P1/B4) had 25 asymptomatic leaves. Molecular analyses confirmed the presence of GRSV in branches P1/B1 and P1/B2. Soon after the branches were cut from the plant and placed in pots for rooting, most of the leaves senesced and died, leaving only a few asymptomatic leaves on each branch.

After six months of vegetative development, the symptoms reappeared in the P1/B1 plant. The plants emitted five branches with symptomatic leaves on two of the branches. Symptomatic leaves were observed in the upper thirds of branches 4 and 5 (**Figure 4**). Among the 205 leaves, 189 were asymptomatic and 16 were symptomatic. RT-PCR and RT-qPCR analyses confirmed the presence of GRSV only in the symptomatic leaves of these branches (**Table 5**). The nucleotide sequence (GenBank OK539549) of the amplicon obtained from branch 5 showed 99.30% identity to another GRSV isolate (GenBank KY400110).

**Table 5:** Detection of GRSV by RT-PCR and RT-qPCR in total RNA extracted from symptomatic and asymptomatic leaves and roots of vegetatively propagated *P. pudica* plants.

Plant	Portion analyzed	Leaf Symptoms	RT-PCR	RT-qPCR (CT*)
P1/B1	Leaves of the lower third of branch 1	No	–	- (31)
	Leaves of the middle third of branch 1	No	–	- (31)
	Leaves of the upper third of branch 1	No	–	- (31)
	Leaves of the lower third of branch 2	No	–	- (35)
	Leaves of the middle third of branch 2	No	–	- (35)
	Leaves of the upper third of branch 2	No	–	- (35)
	Leaves of the lower third of branch 3	No	–	- (34)
	Leaves of the middle third of branch 3	No	–	- (34)
	Leaves of the upper third of branch 3	No	–	- (34)
	Leaves of the lower third of branch 4	No	–	nt**
	Leaves of the middle third of branch 4	No	–	nt
	Leaves of the upper third of branch 4	Yes	+	**+ (16)**
	Leaves of the lower third of branch 5	No	–	nt
	Leaves of the middle third of branch 5	No	–	nt
	Leaves of the upper third of branch 5	Yes	+	**+ (15)**
P1/B2.1	Root		–	- (38)
	Leaves of the lower third of branch 1	No	–	- (31)
	Leaves of the middle third of branch 1	No	–	- (31)
	Leaves of the upper third of branch 1	Yes	+	**+ (16)**
	Leaves of the lower third of branch 2	No	–	- (31)
	Leaves of the middle third of branch 2	No	–	- (31)
	Leaves of the upper third of branch 2	Yes	+	**+ (16)**
P1/B2.2	Root		+	**+ (17)**
	Leaves of the lower third of branch 1	No	–	- (37)
	Leaves of the middle third of branch 1	No	–	- (37)
	Leaves of the upper third of branch 1	No	–	- (37)

* CT, cycle threshold; ** nt, not tested.

After seven months (February/2021) of vegetative development, the symptoms reappeared in the P1/B2.1 plant. It had two branches with 93 asymptomatic and ten symptomatic leaves (**Figure 4**). The presence of the virus in symptomatic leaves was confirmed by molecular analysis. GRSV was also detected in samples collected from the root system (**Table 5**). The nucleotide sequence (GenBank OK539550) of the amplicon obtained from the root tissues showed 99.30% identity with another GRSV isolate (GenBank KY400110). The P1/B2.1 plant was maintained in a greenhouse to monitor symptoms from March to November 2021. No new leaves emitted during this period exhibited symptoms of viral infection. RT-PCR confirmed the absence of GRSV in young leaves.

The P1/B2.2 plant was maintained in a greenhouse from July 2020 to November 2021 and showed no symptoms of viral infection (**Figure 4**). Analyses performed to detect GRSV by RT-PCR and qRT-PCR confirmed the absence of the virus (**Table 5**).

The symptoms induced by GRSV in the matrix plant P1 (**Figure 1A**) appeared six months after removing symptomatic branches for vegetative propagation. Symptoms were limited to seven leaves from three branches of more than 400 asymptomatic leaves.

P1/B1 plants were used to evaluate the movement and expression of GRSV symptoms after a new vegetative propagation cycle. The five branches (P1/B1.1 to P1/B1.5) were cut and placed in pots for rooting and vegetative development. The P1/B1 plant was pruned at its base to monitor the symptoms and presence of GRSV in the new shoots. After five months, symptoms of chlorotic lesions appeared on four leaves of P1/B1.3 plant, and RT-PCR and RT-qPCR confirmed the presence of GRSV. GRSV was not detected in P1/B1.1, P1/B1.4, and P1/B1.5 plants. The P1/B1.2 plant died during vegetative development. The P1/B1 plant, which was pruned at the base, showed symptomatic leaves after six months of development. Symptoms were observed in the leaves in the middle third of the plant. The presence of the virus in these leaves was confirmed by RT-PCR and RT-qPCR (**Figure 4**; **Table 4**).

## 4 Discussion

Ninety-six plants of *P. pudica* from different municipalities in Brazil were inspected for symptoms caused by a virus. Only six symptomatic plants were found (**Table 2**). Analyses of ultrathin sections of symptomatic leaf tissue, RT-PCR, RT-qPCR, and nucleotide sequencing confirmed that only the six symptomatic plants were infected with GRSV. Detailed evaluations of symptom manifestation in naturally infected plants and those obtained through vegetative propagation indicated that they were intermittent and restricted to a few leaves of one or a few branches. The symptoms were predominantly confined to some branches’ lower and middle leaves, and their subsequent development resulted in their remission. However, after approximately 5-7 months, a new manifestation of GRSV symptoms occurred in some branches, indicating that the virus remained systemic in the plants. The presence of GRSV in the root system was detected in only one of the analyzed plants (**Table 5**). Furthermore, incomplete particles were observed in the cytoplasm of many cells. These data suggest that plumeria plants exhibit a high level of resistance to natural infection and, primarily, to the systemic movement of GRSV.

Resistance to orthotospovirus infection is a known and studied characteristic of the tomato spotted wilt virus (TSWV)/tomato (*S. lycopersicum*) pathosystem and is governed by the *Sw-5* gene (Zhu et al., 2019). Resistance to systemic invasion accompanied by symptom remission is a less common phenomenon in plant viruses. It was reported for the pathosystem cucumber mosaic virus (CMV, family *Bromoviridae*, genus *Cucumovirus*) and passion fruit plants (*Passiflora edulis*) (Gioria et al., 2002). Naturally infected passionfruit plants showed symptoms in leaves located in limited areas of the stems, the intensity of which decreased toward the newly developed leaves on the stems. Biological, molecular, and serological tests revealed that CMV was present only in symptomatic leaves. Furthermore, vegetatively propagated stems containing symptomatic leaves analyzed for a year after rooting showed symptom remission. CMV was no longer detected in the leaf tissues and root systems of the passionfruit plants. However, the cause of this phenomenon remains unclear.

An irregular distribution of viruses in plants was found in the pathosystems of tomato ringspot virus (TmRSV, family *Secoviridae*, genus *Nepovirus*) and apple plants (*Malus domestica*) by Bitterlin et al. (1984). They pointed out that the location and mode of inoculation influenced the systemic movement of the virus in trees. Translocation of TmRSV occurred slowly with greater efficiency up from the point of inoculation. The virus was predominantly detected in the leaves, followed by samples collected from the bark, and less frequently in the root system. In the present study, GRSV was predominantly detected in samples collected from symptomatic leaf blades. Only one analyzed sample contained the virus detected in the root tissues (**Tables 3**, **5**).

Symptom remission in virus-infected plants is usually caused by the activation of defense mechanisms, such as RNA silencing (Ghoshal and Sanfaçon, 2015). Hull (2009) suggested that symptoms induced by viruses can exhibit cyclical characteristics. This can occur concomitantly with increasing and decreasing viral concentrations in host tissues. Symptom remission may reflect changes in the balance between host-activated RNA silencing and the ability of the virus to overcome this defense mechanism, mainly through RNA-silencing suppressor proteins (Hull, 2009). Further studies are necessary to verify whether RNA silencing is one of the mechanisms responsible for symptom remission during the interaction between GRSV and *P. pudica*.

In transmission electron microscopy, the analyses of ultrathin sections of symptomatic leaf tissue from *P. pudica* revealed the presence of few complete GRSV particles and many dense mass aggregates composed of non-enveloped viral nucleocapsids, suggesting that the virus is morphologically incomplete in the host’s cells. The presence of incomplete orthotospovirus particles was first reported by Ie (1982) for TSWV isolates from *Campanula isophylla* and *N. tabacum*. After successive mechanical transmission to these plants, it was found that instead of complete viral particles, there was a predominance of dense, dark masses between ribosomes devoid of membranes. In the case of two other TSWV isolates from *Amaryllis* sp. and *Amaryllis* × *Nerine*, abundant intact particles and the same dark, dense masses were found after successive mechanical passages.

Verkleij and Peters (1983) studied the biochemical and serological properties of one of these defective TSWV isolates and failed to detect protein 4 (G*n* and G*c* glycoproteins) in the extracts from infected plants. They also found that among the three RNA molecules that make up the TSWV genome, RNA 2 was smaller than that found in plants with standard virus particles. They pointed out that non-production of protein 4 would be associated with deletions in RNA 2, which would provide information for its synthesis. For the GRSV isolate found in *P. pudica* plants, the complete nucleotide sequencing of RNA-M indicated that it encodes G*n* and G*c* glycoproteins. The open reading frame was located between nucleotides 4801-1397. Upon translation, the protein showed 98.59% amino acid identity to the corresponding sequence of the glycoprotein precursor of a GRSV isolate (GenBank AST36118.1) identified in infected peanut plants from Brazil. Verkleij and Peters (1983) suggested that the host plant is essential for generating defective TSWV particles. Although aggregates of a dense mass composed of unenveloped viral nucleocapsids have been found primarily after successive mechanical transmission of the virus in host plants, it cannot be ruled out that they occur after natural transmission of the virus in plants through thrip vectors, as is the case in nature-infected *P. pudica* plants.

In turn, it is known that the nucleocapsids of orthotospoviruses lacking an envelope are not transmitted by thrips, as demonstrated by Nagata et al. (2000) for a TSWV mutant and the thrips *Frankliniella occidentalis*. The authors concluded that the TSWV envelope contains determining factors for the virus to infect thrips cells and later transmit to plants. Thus, the transmission of GRSV between *P. pudica* plants by thrips may be hampered by the presence of many nucleocapsids lacking an envelope. Transmission experiments of GRSV to *P. pudica* plants by thrips are needed to confirm this hypothesis.

To our knowledge, this is the first report of a viral infection in *P. pudica* plants. The low incidence of GRSV-infected *P. pudica* plants in nature is associated with resistance to viral infection, and the limitations of systemic movement and expression of symptoms in infected plants suggest that this orthotospovirus does not pose a threat to this ornamental plant. *P. pudica* plants can be propagated using seeds or vegetation (Chamakuri et al., 2020). The use of seeds or vegetative propagation of asymptomatic branches from infected plants, followed by virus indexing, could be an alternative method for obtaining GRSV-free *P. pudica* plants.

## Data availability statement

The datasets presented in this study can be found in online repositories. The names of the repository/repositories and accession number(s) can be found below: https://www.ncbi.nlm.nih.gov/genbank/, OQ656765 https://www.ncbi.nlm.nih.gov/genbank/, OQ656766 https://www.ncbi.nlm.nih.gov/genbank/, OQ656767 https://www.ncbi.nlm.nih.gov/genbank/, OK539546 https://www.ncbi.nlm.nih.gov/genbank/, OK539547 https://www.ncbi.nlm.nih.gov/genbank/, OM972900 https://www.ncbi.nlm.nih.gov/genbank/, OK539548 https://www.ncbi.nlm.nih.gov/genbank/, OM972901 https://www.ncbi.nlm.nih.gov/genbank/, OM972902.

## Author contributions

Study conception and design: GF, FO, and JR. Data collection: GF, FO, HK, EC, EK, and JR. Analysis and interpretation of results: GF, FO, CF, VB, MR-J, RK-S, EK, and JR. Draft manuscript preparation: GF and JR. All authors contributed to the article and approved the submitted version.

## Supplementary material

Supplementary Figure 1 Coverage of the mapped reads to the reference sequences corresponding to the **(A)** L (GenBank MH742956), **(B)** M (GenBank MH742957), and **(C)** S (GenBank MH742958) RNA segments of an isolate of the orthotospovirus groundnut ringspot virus (GRSV). The mapping statistics are presented in the right-hand gray panel of each figure.
