# Supplementary figures and images for: Infection of groundnut ringspot virus in Plumeria pudica characterized by irregular virus distribution and intermittent expression of symptoms
Source: Front Plant Sci. 2023 Jul 26;14:1202139. doi: 10.3389/fpls.2023.1202139 (PMC10410559; doi:10.3389/fpls.2023.1202139)

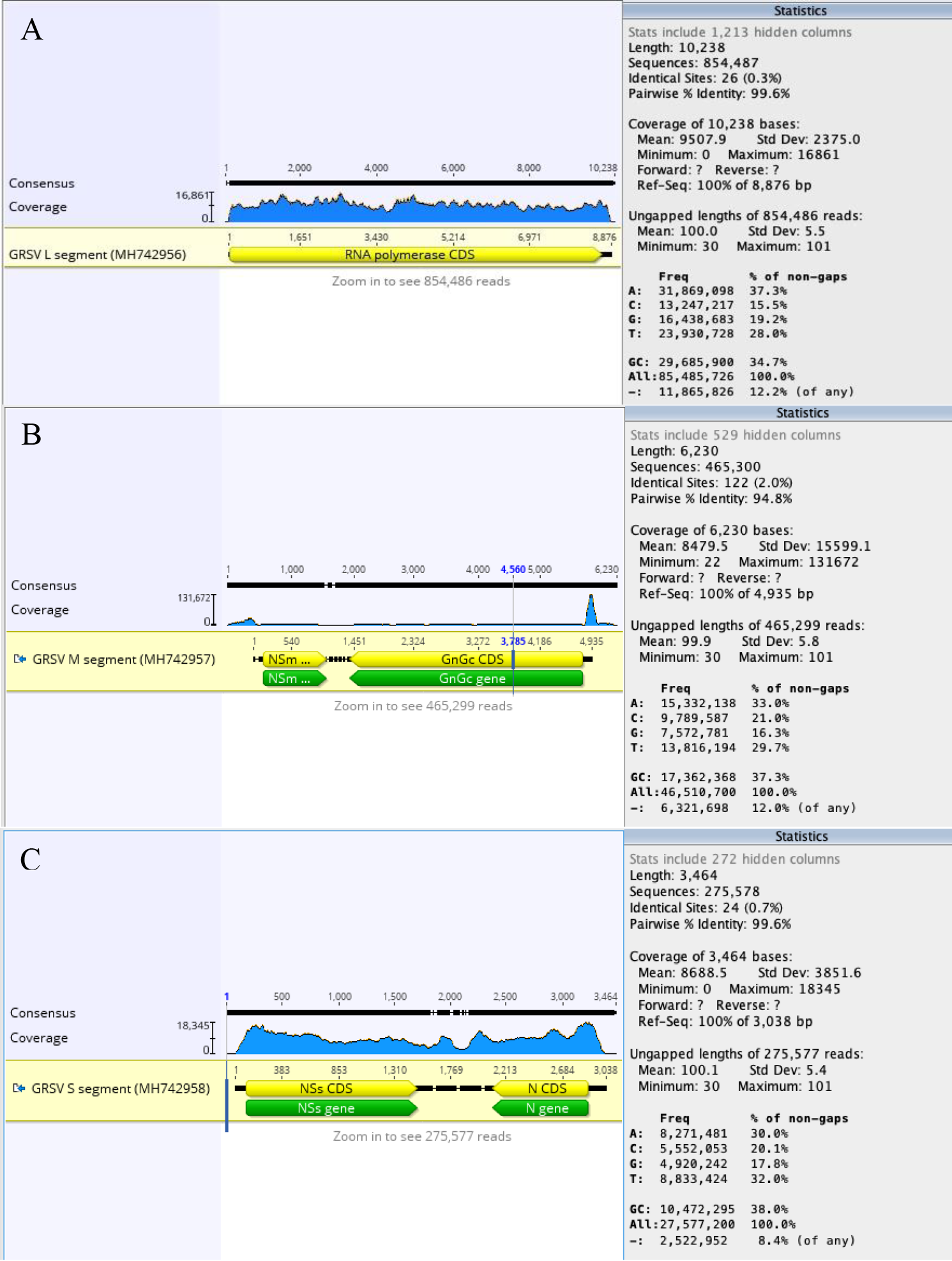

Supplement: Supplementary Figure 1 — Coverage of the mapped reads to the reference sequences corresponding to the (A) L (GenBank MH742956), (B) M (GenBank MH742957), and (C) S (GenBank MH742958) RNA segments of an isolate of the orthotospovirus groundnut ringspot virus (GRSV). The mapping statistics are presented in the right-hand gray panel of each figure. [file Image_1.tif]
